# Selection markers for transformation of the sequenced reference monokaryon Okayama 7/#130 and homokaryon AmutBmut of *Coprinopsis cinerea*

**DOI:** 10.1186/s40694-020-00105-0

**Published:** 2020-10-12

**Authors:** Bastian Dörnte, Can Peng, Zemin Fang, Aysha Kamran, Cut Yulvizar, Ursula Kües

**Affiliations:** 1 Molecular Wood Biotechnology and Technical Mycology, Büsgen-Institute, University of Goettingen, Büsgenweg 2, 37077 Goettingen, Germany; 2 Goettingen Center for Molecular Biosciences (GZMB), University of Goettingen, Goettingen, Germany; 3 School of Life Sciences, Anhui University, Hefei, 230601 China; 4 Anhui Key Laboratory of Modern Biomanufacturing, Hefei, 230601 China; 5 Present Address: Institute for Microbiology and Genetics, University of Goettingen, 37077 Goettingen, Germany

**Keywords:** Adenine auxotrophy, De novo purine biosynthesis, Transformation vector, Para-aminobenzoic acid-auxotrophy, Tryptophan auxotrophy, Hygromycin B resistance, Basidiomycete

## Abstract

**Background:**

Two reference strains have been sequenced from the mushroom *Coprinopsis cinerea*, monokaryon Okayama 7/#130 (OK130) and the self-compatible homokaryon AmutBmut. An adenine-auxotrophy in OK130 (*ade8-1*) and a *para*-aminobenzoic acid (PABA)-auxotrophy in AmutBmut (*pab1-1*) offer selection markers for transformations. Of these two strains, homokaryon AmutBmut had been transformed before to PABA-prototrophy and with the bacterial hygromycin resistance marker *hph*, respectively.

**Results:**

Gene *ade8* encodes a bifunctional enzyme with an N-terminal glycinamide ribonucleotide synthase (GARS) and a C-terminal aminoimidazole ribonucleotide synthase (AIRS) domain required for steps 2 and 5 in the de novo biosynthesis of purines, respectively. In OK130, a missense mutation in *ade8-1* rendered residue N231 for ribose recognition by the A loop of the GARS domain into D231. The new *ade8*^+^ vector p*Cc*Ade8 complements the auxotrophy of OK130 in transformations. Transformation rates with p*Cc*Ade8 in single-vector and co-transformations with *ade8*^+^-selection were similarly high, unlike for *trp1*^+^ plasmids which exhibit suicidal feedback-effects in single-vector transformations with complementation of tryptophan synthase defects. As various other plasmids, unselected p*Cc*Ade8 helped in co-transformations of *trp1* strains with a *trp1*^+^-selection vector to overcome suicidal effects by transferred *trp1*^+^. Co-transformation rates of p*Cc*Ade8 in OK130 under adenine selection with nuclear integration of unselected DNA were as high as 80% of clones. Co-transformation rates of expressed genes reached 26–42% for various laccase genes and up to 67% with *lcc9* silencing vectors. The bacterial gene *hph* can also be used as another, albeit less efficient, selection marker for OK130 transformants, but with similarly high co-transformation rates. We further show that the *pab1-1* defect in AmutBmut is due to a missense mutation which changed the conserved PIKGT motif for chorismate binding in the C-terminal PabB domain to PIEGT in the mutated 4-amino-4-deoxychorismate synthase.

**Conclusions:**

*ade8-1* and *pab1-1* auxotrophic defects in *C. cinerea* reference strains OK130 and AmutBmut for complementation in transformation are described. p*Cc*Ade8 is a new transformation vector useful for selection in single and co-transformations of the sequenced monokaryon OK130 which was transformed for the first time. The bacterial gene *hph* can also be used as an additional selection marker in OK130, making in combination with *ade8*^+^ successive rounds of transformation possible.

## Background

*Coprinopsis cinerea* is a well-known model fungus for studying biological processes in Agaricomycetes. As early as in 1987 and for one of the first fungi of all, protoplast transformation of *C. cinerea* was successfully established by Binninger et al. [1]. For DNA transformation, protoplasts are usually generated from easy to regenerate single-celled haploid aerial mitotic spores (oidia) and are commonly treated in PEG 4000/CaCl_2_-mediated cold-shock transformation with ca. 1 µg plasmid DNA. The protocol is highly efficient with in best cases up to several hundreds of transformants per µg DNA [1–4]. Up till today, the protoplasting and transformation protocol of Binninger et al. [1] has not much been changed in the principles. However, the method was later more simplified and specified in details as compared to the original description [2, 3]. Comprehensive troubleshooting tips have been provided to identify and correct possible subconscious while crucial small handling errors in order to ensure reliable transformation [4].

One reason for the very high transformation rates of *C. cinerea* is that mostly homologous selection markers are used for the complementation of auxotrophies. The bifunctional tryptophan synthase gene *trp1*^+^ cloned in the pUC9-based 9.8 kb-sized plasmid pCc1001 [1] is so far most often applied in transformation. More recently, the shorter pBluescript KS^−^-based *trp1*^+^-plasmid pBD5 (7 kb) with higher copy number in *Escherichia coli* and the *trp1*^+^ yeast-shuttle vector pYtrp1 (9.9 kb) have been established [5]. The two gene halves of *trp1*^+^, *i.e. trpA*^+^ for the Trp1 A domain responsible for the aldo-cleavage of indole-3-glycerol-phosphate (IGP) into indole and *trpB*^+^ for the Trp1 B domain for the subsequent pyridoxal phosphate cofactor-dependent conversion of indole with serine to tryptophan [5], have been functionally separated into individual yeast-shuttle vectors pYAdom (8.3 kb) and pYBdom (8.7 kb) to allow successive rounds of transformation into *C. cinerea trp1.1,1.6* double mutant strains with first *trp1.6* (*trpB*) and then *trp1.1* (*trpA*) complementation [6].

**Fig. 1:**
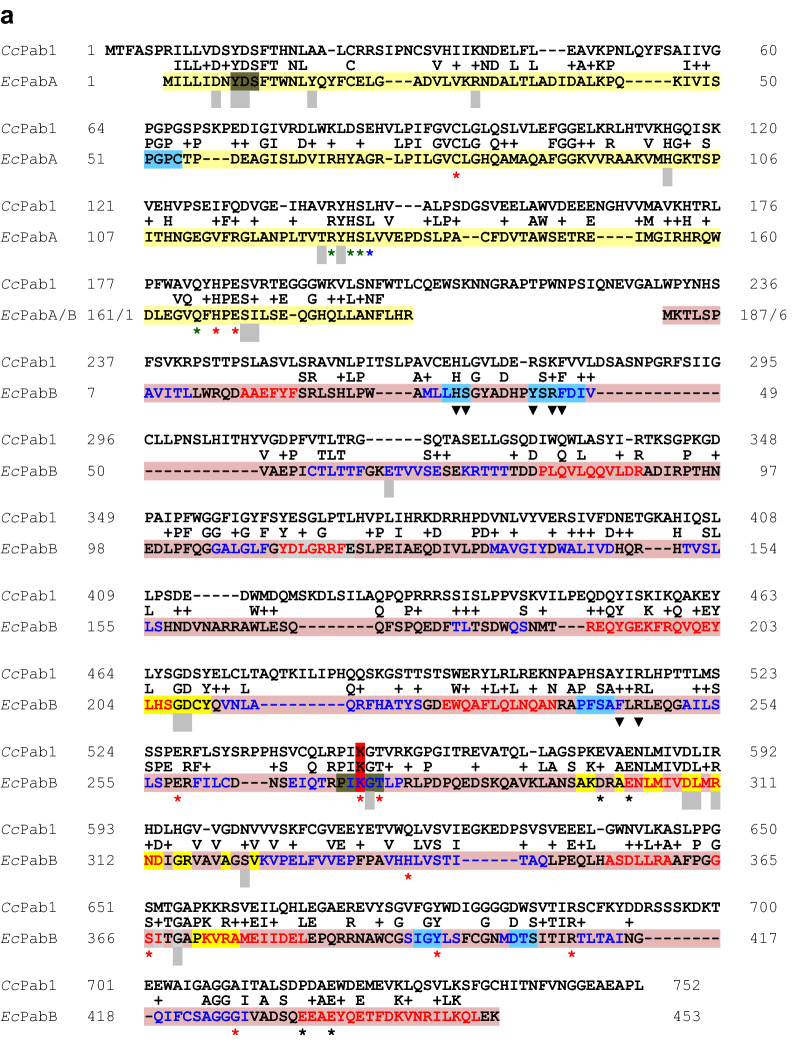

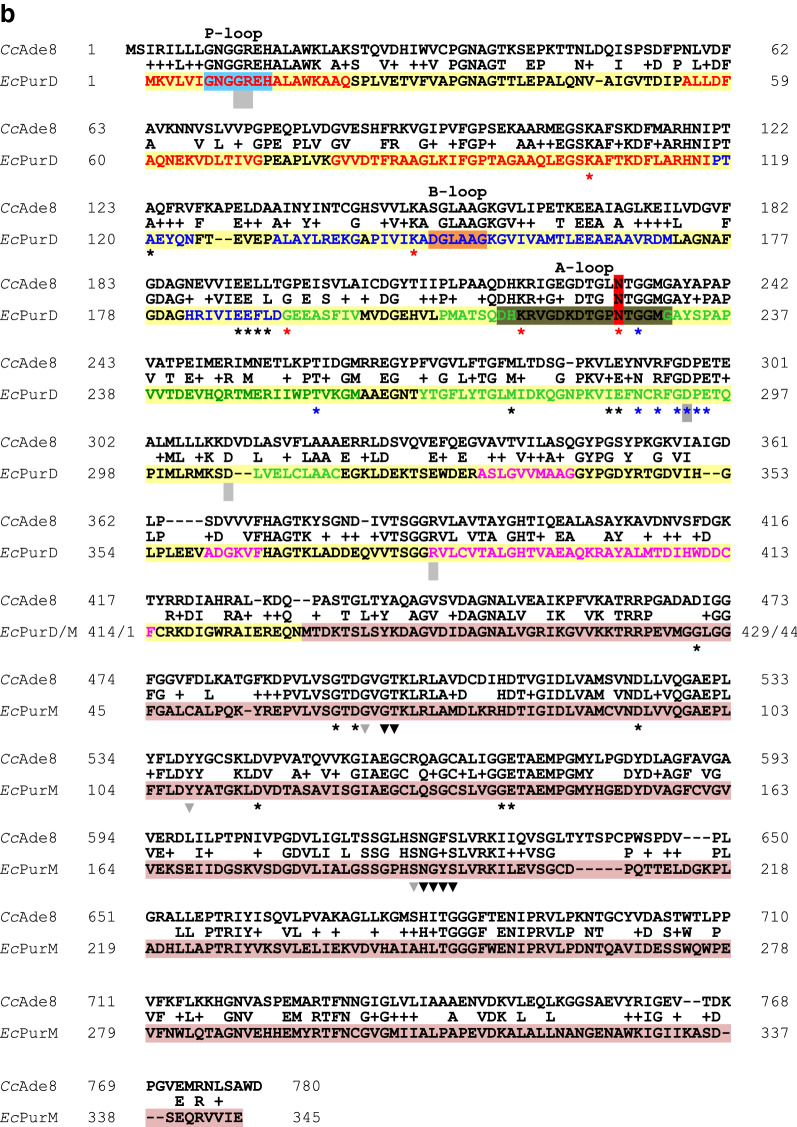
Alignment of A. wt Pab1 from *C. cinerea* monokaryon OK130 (*Cc*Pab1) with PabA (*Ec*PabA, underlaid in yellow) and PabB of *E. coli* (*Ec*PabB, underlaid in dusky pink) and B. wt Ade8 from *C. cinerea* strain AmutBmut (*Cc*Ade8) with PurD (*Ec*PurD, underlaid in yellow) and PurM of *E. coli* (*Ec*PurM, underlaid in dusky pink), respectively. **a** The catalytic triad, glutamine binding residues and residues involved in ammonia tunnel formation in PabA are marked with red, green and blue symbols *, respectively. Other residues affecting enzymatic activities and bonding to PabB are marked with grey squares. The position of a stabilizing residue stretch called oxyanion hole is underlaid in light blue, a sequence stretch for chorismate signal transfer in olive [29, 30, 75]. Red letters in PabB mark helical regions, blue letters β-sheets. The conserved PIKGT motif, sequences for interaction with PabA, for signal transfer of chorismate binding, and of a binding pocket for tryptophan implicated in structural stabilization are underlaid in olive, bright yellow, grey and light blue, respectively. The residue K in the PIKGT motif which is mutated in *C. cinerea* AmutBmut (K546E) is marked in red. Symbols * in red and black mark (predicted) active site residues and Mg^2+^-binding residues in two chorismate-interacting helices, respectively. Triangles in black indicate residues that contact the bound tryptophan and grey squares further residues where mutations affect functionality [28–31, 76]. **b** Red, blue, green and magenta letters mark the N, B, A, and C domains of PurD. The positions of the P-loop and the flexible A and B loops in PurD [56] are underlaid in light blue, olive and orange, respectively. Symbols * in black, red, and blue mark residues that recognize the adenine base, ribose and phosphate of the nucleotide, whereas grey squares indicate residues interacting with the ligand PRA [56, 57]. The residue N in the A loop which is mutated in *C. cinerea* OK130 (N231D) is marked in red. In PurM, symbols * mark (predicted) nucleotide binding residues and triangles (in grey predicted) binding sites of the substrate *N*-formylglycinamidine ribonucleotide (FGAM) [58]

Two other genes from the tryptophan biosynthesis pathway cloned in vectors for transformation of suitable *C. cinerea* mutant strains are *trp2*^+^ [2] for a trifunctional enzyme with glutamine amidotransferase (GATase; anthranilate synthase component II which releases ammonia from glutamine), phosphoribosylanthranilate isomerase (PRAI) and indol-3-glycerol-phosphate synthase (IGPS) activities [5], and the gene *trp3*^+^ [7, 8] for anthranilate synthase component I which uses ammonia and chorismate to produce anthranilate, 2-aminobenzoic acid [5]. Cloned is also a positively selectable mutant gene *trp3*^*iar*^ for a dominant 5-fluoroindole-resistant anthranilate synthase component I mutant [9]. *pab1*^+^ vectors [3, 10] have been provided for complementation of auxotrophies in *para*-aminobenzoic acid (PABA) synthesis caused by defects in the bifunctional enzyme Pab1. Conventionally, this fungal enzyme is known as PABA synthase but more precisely, it is a 4-amino-4-deoxychorismate (ADC) synthase. The enzyme consists of an N-terminal PabA domain (37% identity, 53% similarity to *E. coli* PabA; Fig. 1a) and a C-terminal PabB domain (30% identity, 49% similarity to *E. coli* PabB; Fig. 1a). PabA presents PABA synthase component II (or better called ADC synthase component II) and has a PabB-dependent GATase function. The PabB domain as PABA synthase component I (or more precisely ADC synthase component I) will aminate chorismate in order to yield ADC as the direct precursor of PABA to be formed by an ADC lyase (PabC) [11, 12]. Regarding further functional *C. cinerea* selection markers, a cosmid is mentioned in a conference proceeding that could complement an uncharacterized *ade8* defect of *C. cinerea* in transformation [13].

Selection for dominant resistances is another strategy to obtain transformants. A carboxin resistance selection marker (*sdi1*^*R*^) has been generated by site-specific mutation of the native *C. cinerea sdi1* gene for the iron-sulphur protein subunit (subunit SdhB) of the mitochondrial succinate dehydrogenase (SDH) complex [14]. Flutolanil and carboxin resistance is moreover mediated through a spontaneous point mutation by an allele of the *sdhC* gene for the SdhC cytochrome *b*_*560*_ subunit of the SDH complex [15]. The *sdi1*^*R*^ allele has been cloned behind the heterologous constitutive *gpdII* promoter of *Agaricus bisporus* [14] which is highly active in *C. cinerea* [16]. Transformation rates of such optimized *sdi1*^*R*^ vectors were then high with > 100 transformants/µg plasmid DNA [14]. Transformation rates with the *sdhC* mutant allele under natural regulatory sequences in contrast were low with 1.0 to 4.8 transformants/10^5^ viable protoplasts [15].

As functional bacterial resistance genes in *C. cinerea*, vectors with the *E. coli* hygromycin B phospotransferase gene *hph* [14, 17] and the *Streptoalloteichus hindustanus* gene *ble* for a phleomycin binding protein are available [14]. Insertion of a functional intron after the second codon of the *ble* gene was essential for successful expression of the gene in *C. cinerea* behind the *A. bisporus gpdII* promoter [14]. Regarding expression of *hph*, presence of an intron was not crucial. However, the entire coding region of *hph* is required to be inserted behind an active promoter in *C. cinerea* (native *tub1* promoter or heterologous *A. bisporus gpdII* promoter) [14, 17]. The best-known *hph*-vector pAN7-1 from transformation in filamentous ascomycetes for example lacks the first two codons for two lysine residues and by this reason did not function in *C. cinerea* transformation [14] unlike, although at low frequency (1 to 5 transformants/µg plasmid DNA), in the basidiomycetes *Hebeloma cylindrosporium* [18] and *Crinipellis perniciosa* [19].

The obvious advantage of usage of dominant resistance markers for selection is that transformation becomes independent of any auxotrophies that are needed to be generated. Though, using dominant resistance markers for *C. cinerea* somewhat complicates the transformation procedure. Protoplasts are spread onto regeneration agar but for suppression of unwanted background growth, it requires an extra regeneration agar overlay with antibiotics for selection for positive transformants to grow through this overlay [14, 16]. Handling of complementation of auxotrophies in transformation in contrast is much easier by just plating and then incubating protoplasts on regeneration agar [2–4]. However, through complementation of available auxotrophies and selections for dominant resistance markers, extra rounds of successive transformations in a same background become possible. Such makes strains more versatile for repeated genetic manipulations.

So far, the genomes of two distinct *C. cinerea* strains, the monokaryon Okayama 7/#130 (short OK130) and the self-fertile homokaryon AmutBmut, have been sequenced by the Broad Institute (Boston, MA) and the JGI (Joint Genome Institute, Walnut Creek, CA), respectively [20, 21]. AmutBmut carries a *pab1-1* mutation and is easily be transformed by *pab1*^+^ vectors, a feature which is very useful in studying dikaryon-specific growth behavior and fruiting body development in this self-fertile strain, independently of a second genome [22–24]. On the other hand, to the best of our knowledge, strain OK130 with the first *C. cinerea* reference genome established had not yet been transformed before. This reference monokaryon carries an *ade8-1* mutation [8] which we used here in transformation for selection by complementation. Missense mutations in the defective alleles *pab1-1* and *ade8-1* were identified in this study. In addition, transformants of OK130 were obtained with the dominant bacterial hygromycin resistance selection marker *hph*.

## Results and discussion

### Genes *pab1* and *ade8* in *C. cinerea*

Classical mapping of *C. cinerea* localized gene *pab1* 0.5 cM upstream and gene *ade8* 1.3 cM downstream to the bipartite *A* mating type locus (consisting of *Aα* and *Aβ*) on linkage group I [25, 26]. The ca. 20 kb-long *A43* mating type allele with all its homeodomain transcription factor genes locates at position Chr_1:2,666,138–2,647,809 in the sequenced OK130 genome [20, 27]. *pab1* [11] is found at location Chr_1:2,699,078–2,701,362, 32.94 kb apart from the 3′ end of the closest *A43α* gene *a1-1* [20, 27]. *pab1*^+^ in OK130 (Broad model CC1G_01849T0) distinguishes from the *pab1-1* allele in AmutBmut (JGI ID 414607) by a point mutation in codon 546, with a change from AAG to GAG. This missense mutation resulted in a K546E exchange in the PabB domain within the highly conserved ADC synthase component I motif PIKGT. Lysine in the wildtype (wt) covalently binds to the C_2_ of chorismate to initiate with the ammonia-group of glutamine the enzymatic formation of ADC ([28–31], Fig. 1a).

The recombination rate between *pab1* and *Aα* calculates as ≥ 66 kb/cM (≥ 70-75 kb/cM with the whole *pab1* gene sequence included [8, 32]). Other studies estimated the average recombination frequency over the *C. cinerea* genome higher as 27.9 kb/cM [33] and 33 kb/cM [20], respectively. With the same kb/map unit relations, *ade8* should then locate about 40 to 100 kb downstream of *Aβ*. A gene for a bifunctional purine biosynthetic protein (CC1G_01782T0; Table 1) was found in the OK130 genome at location Chr_1:2,548,109–2,550,858, 97 kb downstream to the closest *A43β* gene *d1-1* [20, 27], with a possible recombination rate of 74.6 kb/cM using 1.3 cM for calculation.

**Table 1:** Identification of gene functions in de novo purine biosynthesis, formation of folates and THF-mediated one-carbon metabolism in *C. cinerea* OK130

Steps in de novo purine synthesis and interlinked processes		Enzyme			
		Name, GenBank accession number		*C. cinerea* OK130	
Substrate—product	Enzymatic function	*E. coli*	*S. cerevisiae*	Broad model, classic name	Chromosomal location in OK130*
PRPP to PRA	Glutamine amidophosphoribosyltransferase (GPAT)	PurF, CAA30971	Ade4, P04046	CC1G_01222T0, likely Ade2	Chr_2:1,228,139–1,230,457
PRA to GAR	Glycinamide ribonucleotide synthase (GARS)	PurD, CAA36213	N-terminal domain of bifunctional Ade5,7, NP_011280	CC1G_01782T0, N-terminal domain of bifunctional Ade8	Chr_1:2,548,109–2,550,858
GAR to FGAR	Phosphoribosylglycinamide formyltransferase (GART)	PurN, P08179	Ade8, NP_010696	CC1G_04353T0, potentially Ade4	Chr_1:715,850–716,603
	[Bacterial alternative: formate-dependent phosphoribosylglycinamide formyltransferase]	PurT, NP_416363	–	–	–
FGAR to FGAM	Phosphoribosylformylglycinamidine synthase (FGAMS)	PurL, THH53207	Ade6, NP_011575	CC1G_11804T0, potentially Ade4	Chr_6:3,409,097–3,413,188
FGAM to AIR	Aminoimidazole ribonucleotide synthase (AIRS)	PurM, THH44093	C-terminal domain of bifunctional Ade5,7, NP_011280	CC1G_01782T0, C-terminal domain of bifunctional Ade8	Chr_1:2,548,109–2,550,858
AIR to CAIR	5-(Carboxyamino)imidazole ribonucleotide synthase + 5-(carboxyamino)imidazole ribonucleotide mutase (AIR carboxylase)	PurK + PurE, NP_415055, NP_415056	Fused Ade2, P21264	CC1G_11091T0, fused Ade1	Chr_5:473,822–471,864
CAIR to SAICAR	Phosphoribosylaminoimidazole-succinocarboxamide synthase (SAICARS)	PurC, NP_416971	Ade1, NP_009409	CC1G_05887T0	Chr_7:2,536,570–2,535,540
SAICAR to AICAR	Adenylosuccinate lyase	Bifunctional PurB, THI73349	Bifunctional Ade13, NP_013463	CC1G_08733T0, bifunctional Ade5	Chr_10:936,450–934,462
AICAR to FAICAR	AICAR transformylase	Bifunctional PurH, NP_418434	Bifunctional Ade16, NP_009409 or isoenzyme Ade17, NP_013839	CC1G_08365T0	Chr_7:2,467,163–2,464,958
FAICAR to IMP	IMP cyclohydrolase				
IMP to SAMP	Adenylosuccinate synthase	PurA, NP_418598	Ade12, NP_014179	CC1G_10072T0	Chr_2:407,487–405,875
SAMP to AMP	Adenylosuccinate lyase	Bifunctional PurB, THI73349	Bifunctional Ade13, NP_013463	CC1G_08733T0, bifunctional Ade5	Chr_10:936,450–934,462
GTP to DHNTP	GTP cyclohydrolase	FolE, NP_416658	Fol2, P51601	CC1G_14672T0	Chr_5:2,160,832–2,161,846
DHNTP and PABA to 7,8-DHP to DHF	Trifunctional dihydropteroate synthase/dihydrohydroxymethylpterin pyrophosphokinase/dihydroneopterin aldolase	FolB + FolK + FolP, NP_417530, 3IP0_A, NP_417644	Fused Fol1, NP_014143	CC1G_15556T0, fused	Chr_6:783,810–781,706
DHP to DHF	Dihydrofolate synthase/ folylpolyglutamate synthase	FolC, P08192	Fol3, NP_013831	CC1G_00421T0	Chr_2:3,461,586–3,463,459
			Met7, NP_014884	CC1G_04850T0	Chr_5:1,857,755–1,855,944
DHF to THF	Dihydrofolate reductase	FolA, 4GH8_A	Dfr1, P07807	CC1G_012670T0, potentially Ade9	Chr_1:1,571,610–1,572,294
5,10-Methylene-THF to 10-formyl-THF	NADP-dependent methylentetrahydrofolate cyclohydrolase, methylenetetrahydrofolate dehydrogenase	Bifunctional FolD, 5O22_D	N-terminal domain of trifunctional Ade3, NP_011720	CC1G_13910T0, N-terminal domain of trifunctional enzyme	Chr_2:1,522,272–1,525,659
	NAD^+^-dependent methylenetetrahydrofolate dehydrogenase		Mtd1, Q02046	CC1G_01428T0	Chr_5:2,438,251–2,463,749
10-Formyl-THF to formate and THF	Formyltetrahydrofolate deformylase	PurU, THH46545	–	–	–
3-PHP to phosphoserine	*O*-Phospho-L-serine:2-oxoglutarate aminotransferase	SerC, THI65673	Ade9 = Ser1, NP_014827	CC1G_11497T0	Chr_2:2,589,569–2,588,293
L-serine to glycine + THF to 5,10-CH_2_-THF	Glycine/serine hydroxymethyltransferase	SHMT, 3G6M_A	SHM2, NP_013159	CC1G_10328T0	Chr_6:1,087,903–1,089,686

*Assigning classical linkage groups [50–52] and adenine auxotrophies [49, 50] to the new chromosome classification in OK130 sorted after sequence length [20]: Chromosome 1 = classical linkage group I with *A* mating type locus, *ade8* (with function prior to AIR ring closure [49]) and, 9 cM away from the *A* mating type locus, *ade9* [51, 52] which appears to function as a regulatory enzyme rather than within the direct de novo pathway of purine biosynthesis [49] and might therefore be a dihydrofolate reductase gene for THF production located 752 kb downstream to *A43β* (recombination rate is then 83 kb/cM) with potential cross-pathway effects between de novo purine biosynthesis and THF-mediated C1, histidine and methionine metabolisms [42, 46]. A gene with potential GART function (step 3 in de novo purine biosynthesis) as one candidate for the unmapped gene *ade4* functioning in the pathway prior to imidazole ring closure [49, 52] is present 1932 kb downstream of *A43β*, closer to the telomere. Chromosome 2 = classical linkage group III with *trp1*, *trp3*, *ade2* (with function prior to AIR ring closure [49]) and *ade12* (0.2 cM apart from *ade2* [52] = an estimated distance of 5.6 to 6.6 kb [20, 33] which could point to CC1G_01221T0 for *S*-adenosylmethionine synthase at position Chr_2:1,226,385–1,227,850 or CC1G_01223T0 for diadenosine polyphosphate hydrolase and related proteins of the histidine triad (HIT) family at position Chr_2: 1,231,397–1,230,670 as potential candidates for *ade12*). Chromosome 3 = classical linkage group G with *trp2* [51, 52], *pcc1* [33], and, 16 cM distal to *trp2* [51, 52], *ade3* unidentified here with a function prior to AIR ring closure [49]. Chromosome 5 = classical linkage group IV with *ade1* with CAIR synthase function [49]. Chromosome 6 (with a gene for a FGAMS function as another *ade4* candidate) and chromosome 7 = classical linkage groups unclear. Chromosome 10 = classical linkage group II with *B* mating type locus, the bifunctional *ade5* with adenylosuccinate lyase function [49], *ad/his-1* and *ad/his-2* which are likely *ade5* alleles with cross-pathway effects on histidine biosynthesis via effects of the regulatory metabolite AICAR [46, 49]. Classical linkage groups V and VI with *ade6* and an *ad/met* locus, respectively [51, 52] = new chromosome numbers unclear

Many mutations leading to adenine-auxotrophies belong directly to the de novo purine biosynthesis pathway [34–36]. Other indirect mutations include defects in tetrahydrofolate (THF) cofactor formation, further folate metabolism and THF-mediated C1-metabolism, as well as defects in cross-pathway regulation of de novo purine biosynthesis and syntheses of amino acids (histidine, methionine) mediated by feedback control of certain metabolites [5´-phosphoribosyl-5-monophosphate (AICAR)] or shared transcriptional regulators [35, 37–48]. We screened the OK130 genome for such genes, using known *E. coli* and *Saccharomyces cerevisiae* proteins in tblastn searches. Spread over 7 chromosomes, genes for all enzymatic functions for de novo purine biosynthesis and for other mentioned functions were found (Table 1). Previously, twelve different *ade* complementation groups have been described in *C. cinerea*, two more mutants that react to adenine and histidine (*ad/his1* and *ad/his2*) and another that reacts alternatively to adenine or methionine (*ad/met*) [49, 50]. Ten of these genes have been mapped onto 7 linkage groups [50–52]. Though, in our analysis only four to possibly seven genes (*ade2*, *ade8*, *ade1*, *ade5*, and possibly *ade4*, *ade9*, and *ade12*) from only four linkage groups could be assigned to specific positions on sequenced chromosomes (Table 1), using as additional information their clearly defined biochemical reactions (cases *ade1*, *ade5* [49]) or approximate positions in the de novo purine biosynthesis pathway (*ade2*, *ade3*, *ade4* and *ade8* all act prior to imidazole ring closure [49]) and/or their linkages (*ade2*, *ade3*, *ade5*, *ade8*, *ade9* and *ade12*) to other unquestionably identifiable gene functions on the classical *C. cinerea* map ( [33, 50–52]; see footnote of Table 1). However, no other convincing candidate for gene *ade8* were found in appropriate distance to the *A* locus on chromosome 1 (Table 1).

The protein encoded by the gene at Chr_1:2,548,109–2,550,858 has been annotated in GenBank (EAU92737.2) as ADE1 [*Coprinopsis cinerea* Okayama 7/#130] which conflicts the traditional *C. cinerea* gene nomenclature. *C. cinerea* gene *ade1* resides on linkage group IV of the fungus [51, 52] which corresponds to chromosome 5 in the OK130 genome assorted by chromosome sequence length ( [20], Table 1). Moreover, Ade1 of *C. cinerea* had been shown in the de novo purine biosynthesis to function in the 6th step directly after 5-aminoimidazole ribonucleotide (AIR) ring closure as phosphoribosylaminoimidazole carboxylase in the formation of 5-amino-4-imidazolecarboxamide ribonucleotide (CAIR) ( [49], Table 1).

**Fig. 2:**
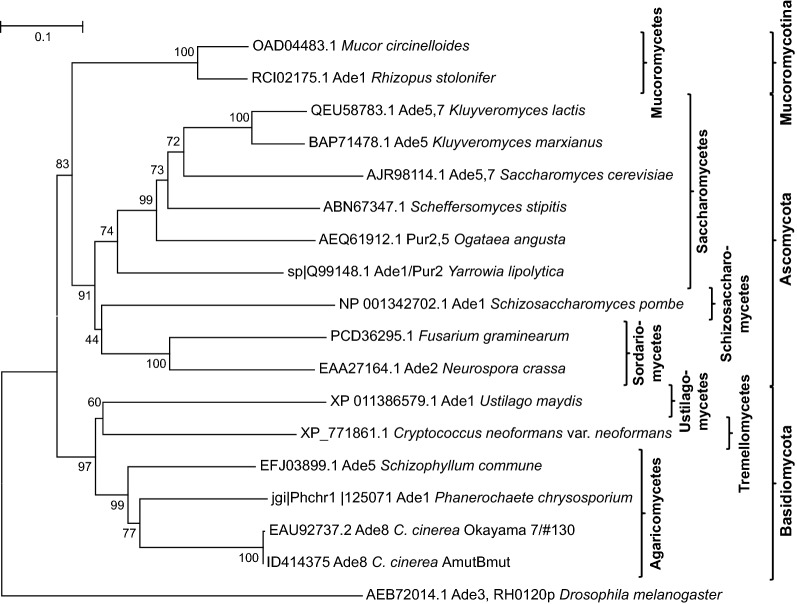
Neighbor-joining phylogenetic tree of bifunctional fungal GARS-AIRS enzymes clustering according to fungal clades. Note that corrections in exon/intron splicing sites have been done for the OK130 Ade8 model (GenBank EAU92737.2 = Broad model CC1G_ 01782T0 = JGI ID 1589), following the RNAseq-supported model for the *ade8*^+^ gene of strain AmutBmut (JGI ID 414375). The *Drosophila melanogaster* Ade3 protein used as outgroup is trifunctional with GARS, AIRS and GART domains, the latter of which was excluded from the analysis

The gene at location Chr_1:2,548,109–2,550,858 has homologs in other fungi that, by historical naming of adenine-auxotrophic mutants, are variably known as *ade1* such as in *Phanerochaete chrysosporium*, *ade5* in *Schizophyllum commune*, *ade2* in *Neurospora crassa*, *ade5,7* in *S. cerevisiae* and *pur2*, *pur2,5* and *pur2,7* in *Yarrowia lipolytica*, *Ogataea angusta* and *Scheffersomyces stipitis*, respectively (Fig. 2). Gene *ade5*^+^ of *S. commune* can complement *ade1* defects of *P. chrysosporium* like the homologous native *ade1*^+^ gene and it can complement *ade2* defects of the ascomycete *N. crassa* [53, 54]. All mentioned fungal genes encode bifunctional enzymes for the de novo biosynthesis of purines, with an N-terminal glycinamide ribonucleotide synthase (GARS) domain and a C-terminal aminoimidazole ribonucleotide synthase (AIRS) domain (Fig. 1b; Table 1) which act in the 2nd and the ring-closing 5th step in de novo purine biosynthesis, respectively [34–36]. *ade5* of *S. commune* and *ade8* of *C. cinerea* are conserved in chromosomal location relative to the position of *Aβ*, similar as their *pab1* genes are relative to *Aα* [8, 32, 55]. The gene for a bifunctional GARS-AIRS enzyme identified here on *C. cinerea* chromosome I with good likelihood thus presents its *ade8* gene.

The N-terminal halves of the fungal bifunctional GARS-AIRS enzymes correspond to bacterial PurD enzymes (49% identity, 67% similarity between the *C. cinerea* enzyme and *E. coli* PurD; Fig. 1b) which are glycinamide ribonucleotide (GAR) synthases represented in structure *e.g.* by the crystalized *E. coli* PurD protein (1GSO_A). PurD catalyzes the 2nd step of the de novo purine biosynthetic pathway, the conversion of phosphoribosylamine (PRA), glycine, and ATP to GAR, ADP (adenosine diphosphate), and phosphate (Pi) ( [35, 56, 57], Table 1). The C-terminal halves of the fungal bifunctional GARS-AIRS enzymes are homologous to bacterial PurM enzymes (55% identity, 67% similarity of the *C. cinerea* enzyme to *E. coli* PurM; Fig. 1b). PurM represented in structure by *E. coli* 1CLI_A is a phosphoribosylformylglycinamidine cyclo-ligase that catalyzes the conversion of formylglycinamide ribonucleotide (FGAM) and ATP to AIR, ADP, and Pi, in the 5th step in de novo purine biosynthesis ( [35, 58], Table 1).

The folded bacterial GARSs consist of the three domains N, A, and C forming the central core of the enzyme and, connected to them by flexible hinges, the outward-extended domain B [56]. Substrate PRA is recognized by specific amino acids in the N, A, and C domains. The A domain further confers the binding site for the ligand glycine ( [56, 57], Fig. 1b). GARSs are members of the ATP-grasp superfamily of enzymes with an atypical ATP-binding site (ATP-grasp fold) comprised by the two domains A and B that catch an ATP between them [59]. Accordingly, the A and B domains primarily define the ATP/ADP binding site of GARSs, with distinct residues in domains A and B and also in N contacting the adenine base, ribose and phosphate, respectively ( [56, 57], please see Fig. 1b for details). Further, the A domain possesses a flexible specific A loop with a highly conserved unique sequence (DHKRVGDKDTGPNTGGMG in *E. coli*, see Fig. 1b) which distinguishes GARSs well from all other members of the ATP-grasp superfamily [56, 57, 59]. Structural analyses of bacterial enzymes revealed N226 in the *E. coli* A loop to recognize ribose [57]. The *E. coli* A loop shares 83–89% sequence identity and 94% sequence similarity with the loops in the fungal enzymes analyzed in Fig. 2, with amino acid N231 of wt *C. cinerea* Ade8 = N226 in PurD of *E. coli* (Fig. 1b). Sequence comparison between the functional *ade8*^+^ copy from AmutBmut and the defective *ade8-1* allele in OK130 revealed a point mutation that altered codon 231 from AAT into GAT and then, within the flexible A loop in the GARS A domain, the highly conserved amino acid N231 into D231 (Fig. 1b). The D231 mutation in the N-terminal GARS half explains then the former observation that Ade8 acts prior to imidazole ring formation [49] and, more specifically, assigns the loss of the Ade8 function in OK130 to the 2nd step of de novo purine biosynthesis.

### The p*Cc*Ade8 vector in fungal transformations

**Fig. 3:**
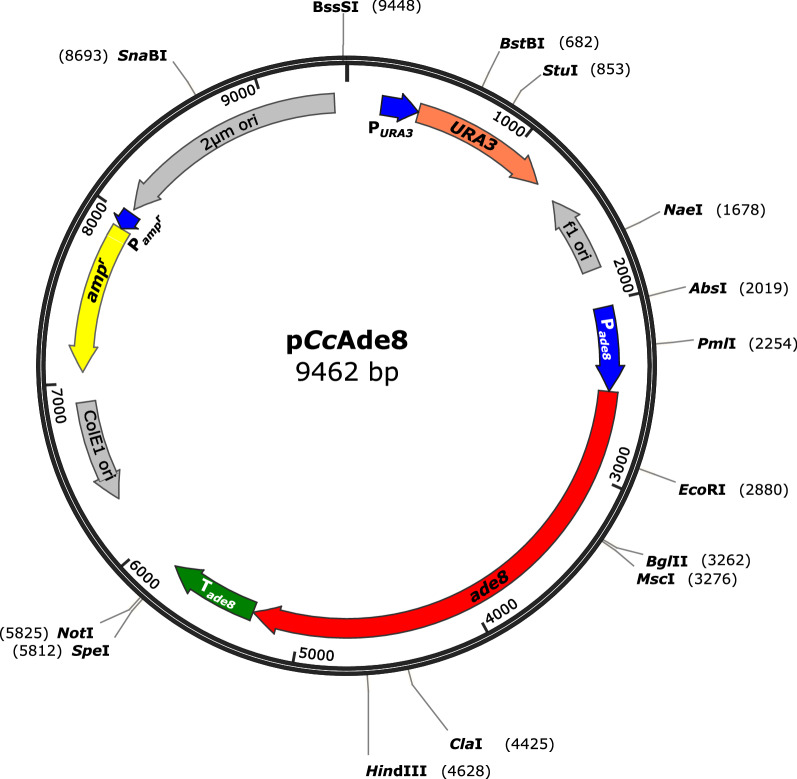
Physical map of the yeast-*E. coli* shuttle vector p*Cc*Ade8 with the cloned *C. cinerea* gene *ade8*^+^

The wt genomic sequence with the *ade8*^+^ coding region (with 9 exons and 8 introns) and 483 and 569 bp upstream and downstream, respectively were PCR-amplified with chimeric primers Ade8f and Ade8r in order to construct vector p*Cc*Ade8 (Fig. 3) by in vivo recombination in yeast with plasmid pRS426 [60]. p*Cc*Ade8 was transformed into monokaryon OK130, alone and, using protoplasts from same batches, in parallel co-transformations with other vectors (Table 2). Adenine prototrophic transformants were selected by growth on adenine-free regeneration agar. Diagnosis PCR with amplicon sequencing verified for 25 transformants randomly chosen from group p*Cc*Ade8 + pYSK-*lcc5* (experiment 1 in Table 2, 1st to 4th day of collection) in all cases the presence and function of the *ade8*^+^ allele.

**Table 2:** Transformations of *C. cinerea* OK130 (*ade8-1*) with *ade8*^+^-vector p*Cc*Ade8 alone or, using same batches of protoplasts, in combination with various pYSK7 laccase gene derivatives

Plasmid(s)	*ade8*^+^-selected transformants collected on*				Total transformants*
	1st day	2nd day	3rd day	4th day	
Experiment 1: Laccase overexpression					
p*Cc*Ade8	17 (1)	15	7	2	41
p*Cc*Ade8 + pYSK7	26 (8)	20 (13)	7 (3)	7 (1)	60 (25)
p*Cc*Ade8 + pYSK-*lcc5*	14 (2)	27 (8)	25 (5)	10 (5)	76 (20)
p*Cc*Ade8 + pYSK-*lcc9*	10 (4)	23 (10)	23 (5)	8 (0)	64 (19)
Experiment 2: Laccase silencing					
p*Cc*Ade8	17	20	12	6	55
p*Cc*Ade8 + pYSK-*lcc9-*antisense-1	5 (2)	7 (2)	12 (6)	6 (4)	30 (14)
p*Cc*Ade8 + pYSK-*lcc9*-antisense-2	2 (1)	9 (5)	17 (12)	8 (6)	36 (24)

*Data in brackets of experiment 1 indicate number of clones with > sixfold increased levels of laccase as detected by activity assay in liquid fermentation and native-PAGE; data in brackets of experiment 2 indicate clones with 2- to 11-fold (2^‒ΔΔCT^) decreases in *lcc9* mRNA transcriptional levels as detected by qRT-PCR

Transformation rates of OK130 to *ade8*^+^ prototrophy in single-plasmid and two-plasmid transformations were in ranges of about 40 to 60 clones each (Table 2). Gene *ade8*^+^ therefore might not confer any significant feedback inhibition on the de novo purine biosynthesis pathway in *C. cinerea*. On the contrary, the *trp1*^+^ selection marker of *C. cinerea* can cause suicidal feedback inhibition on tryptophan biosynthesis with loss of affected clones by a sudden overflow of the amino acid from more expressed *trp1*^+^ copies [5, 6]. This adverse effect on clone viabilities is greater with the single-plasmid transformation than when using mixtures of two plasmids, because singular plasmids in transformation without competition are likely to integrate into twice as many spontaneous DNA breaks per nucleus [5, 6]. As in our previous work with *trp1.1,1.6* monokaryons [5, 6], reduced amounts of tryptophan prototrophs were obtained in only *trp1*^+^-vector pDB5 transformations of strains FA2222 and PG78 as compared to any co-transformations (Tables 3 and 4). p*Cc*Ade8 was newly tested in such co-transformations. Numbers of total transformants under *trp1*^+^ selection were about 1.5–2.5 times higher in the co-transformations with p*Cc*Ade8 than in the single-vector transformation, similar to results of co-transformations with other plasmids (Tables 3 and 4). In co-transformations of monokaryon PG78 with *pab1*^+^-vector pPAB1-2 for selection for PABA-prototrophy, total transformation rates were slightly higher with p*Cc*Ade8 (1.9 × and 1.3x) as compared to other plasmids and in single-plasmid transformation (Table 4). PABA is an intermediate in the biosynthesis of folate [61] which in turn is required in steps of de novo purine biosynthesis for the cofactor THF (Table 1). Co-transforming *pab1*^+^-vector pPAB1-2 with p*Cc*Ade8 might have an initial promoting effect on protoplast regeneration and clone numbers. Typically in transformations of *C. cinerea* with selection schemes other than adenine, we add adenine sulfate as optional supplement to regeneration agar (50 or 100 mg/l) [3, 4] because this can stimulate protoplast regeneration [advice by late L.A. Casselton kindly given to UK].

**Table 3:** Transformations of *C. cinerea* FA2222 (*trp1.1,1.6*) with plasmid pBD5 alone or, using same batches of protoplasts, in combination with other non-directly selectable vectors

Plasmid(s)	*trp1*^+^-selected transformants collected on					Total transformants	Ratio of clones
	1st day	2nd day	3rd day	4th day	5th day		
Experiment 1							
pBD5	13	8	12	3	2	38	1.0
pBD5 + pYSK7*	30 (8)	20 (13)	32 (7)	9 (2)	4 (2)	95 (32)	2.5
pBD5 + pDB3	32	13	25	6	3	79	2.1
pBD5 + pPAB1-2	18	17	17	7	2	61	1.6
pBD5 + p*Cc*Ade8	34	27	11	4	2	78	2.1
Experiment 2							
pBD5	46	38	28	12	11	135	1.0
pBD5 + pYSK7*	94 (31)	89 (22)	68 (27)	15 (12)	5 (3)	271 (95)	2.0
pBD5 + pDB3	69	52	53	15	12	201	1.5
pBD5 + pPAB1-2	76	78	49	28	14	245	1.8
pBD5 + p*Cc*Ade8	100	114	90	26	14	344	2.5

*Date in brackets indicate clones expressing laccases as deduced from stained halos around their colonies. Non-producers of laccase did not stain the agar. Random subsets of unstained pBD5 and of staining pBD5 + pYSK7 clones from both experiments were further tested in liquid fermentations

**Table 4:** Transformations of *C. cinerea* PG78 (*trp1.1,1.6*, *pab1-1*) with either *trp1*^+^ plasmid pBD5 or *pab1*^+^ vector pPAB1-2 alone or, using same batches of protoplasts, in combination with other non-directly selectable vectors

Plasmid(s)	Transformants collected on							Total transformants	Ratio of clones
	1st day	2nd day	3rd day	4th day	5th day	6th day	7th day		
Experiment 1: *trp1*^+^ selection									
pBD5	–	–	–	21	26	14	6	67	1.0
pBD5 + pYSK7	10	16	31	31	14	4	0	106	1.6
pBD5 + pDB3	2	4	0	50	69	15	12	152	2.3
pBD5 + p*Cc*Ade8	–	–	–	45	67	20	16	148	2.2
*pab1*^+^ selection									
pPAB1-2	40	18	14	8	–	–	–	80	1.0
pPAB1-2 + pYSK7	40	13	40	11	–	–	-	104	1.3
pPAB1-2 + pDB3	53	11	31	8	4	–	–	107	1.3
pPAB1-2 + pBD5	14	6	19	13	15	3	–	70	0.9
pPAB1-2 + p*Cc*Ade8	59	32	49	9	3	–	–	152	1.9
Experiment 2: *trp1*^+^ selection									
pBD5	20	21	20	15	7	–	–	83	1.0
pBD5 + pYSK7	26	42	31	13	13	–	–	125	1.5
pBD5 + pDB3	34	38	29	13	7	–	–	121	1.5
pBD5 + p*Cc*Ade8	18	27	49	16	12	–	–	122	1.5
*pab1*^+^ selection									
pPAB1-2	25	29	50	19	13		–	136	1
pPAB1-2 + pYSK7	40	19	37	37	12	–	–	145	1.1
pPAB1-2 + pDB3	33	46	33	26	17	–	–	155	1.1
pPAB1-2 + pBD5	7	30	37	25	18	–	–	117	0.9
pPAB1-2 + p*Cc*Ade8	37	32	54	38	18	–	–	177	1.3

Co-transformation of a selectable vector together with one or more other plasmids is an efficient means to introduce and find non-selectable genes in transformed *C. cinerea* clones [62]. Because we have a deeper interest in laccase functions and applications [16, 63–68], several vectors used here in co-transformations contained either *C. cinerea* laccase genes for enzyme overexpression or were antisense constructs designed for laccase gene silencing (Tables 2 and 3). Most *C. cinerea* monokaryons in fungal cultures have some background laccase activities through expression of Lcc1 and Lcc5 and possibly other enzymes, with the exception of the laccase-free strain FA2222 [16, 64, 65]. Co-transformation to laccase production in monokaryon FA2222 can therefore phenotypically be easily followed up on regeneration agar by enzymatic conversion of the colorless 2,2′-azino-bis (3-ethylbenzothazoline-6-sulfonic acid) (ABTS) into a blue-greenish product seen as well-stained halos around growing clones [16]. Accordingly, co-transformation rates of strain FA2222 with *lcc1* expression vector pYSK7 in this study were 34% and 35%, respectively (Table 3) and were in the range of ratios (25 to 43%) obtained in other *C. cinerea* co-transformation experiments [5, 6, 16]. Each 20 clones were randomly selected for liquid fermentations from the pBD5 and the pBD5 + pYSK7 transformations, respectively. All selected pBD5 transformants showed no enzymatic activity whereas enzymatic activities for the staining pBD5 + pYSK7 transformants were between 0.3 U/mL and 3.4 U/ml.

**Fig. 4:**
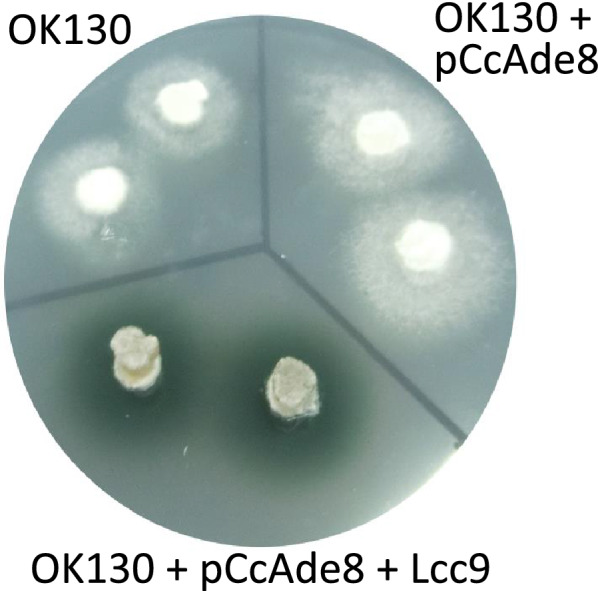
Untransformed *ade8-1* monokaryon OK130 (top left) and p*Cc*Ade8 transformed clones (top right) with barely detectable halos from background laccase activity on ABTS and p*Cc*Ade8 + pYSK-*lcc9* transformants (bottom) with strongly stained broad halos of enzymatically oxidized ABTS. Clones were grown on regeneration agar medium which 0.5 mM ABTS and 50 mg/L adenine sulphate

Monokaryon OK130 typically expresses in cultures some laccase Lcc1 and Lcc5, and traces of Lcc9 [65] why all typical transformants of only p*Cc*Ade8 had faintly stained slender halos around their colonies on medium with ABTS whereas laccase-overexpressing transformants in contrast produced intense broad halos (Table 2; Fig. 4). Co-transformation rates of monokaryon OK130 of selection vector p*Cc*Ade8 with three different laccase overexpression constructs were similar like in the FA2222 co-transformations described above. Co-transformations of monokaryon OK130 led in 26% to 42% of all clones to phenotypically increased enzyme activities, from background laccase activities in OK130 and p*Cc*Ade8 control transformants of around 0.1 U/ml to 0.6–3.1 U/ml for *lcc1* and 2.0–7.5 U/ml for *lcc5* and *lcc9* transformants as determined by activity tests in liquid fermentation and further shown in native-PAGE by strongly increased staining activity of those band which was characteristic for the respective laccase gene used in transformation. Only one clone from single-p*Cc*Ade8 transformation produced sizeable amounts of laccase (2.3 U/ml) by overexpression of both Lcc1 and Lcc5 which was probably caused by an unknown mutation in the clone (experiment 1, Table 2).

In experiment 2 in Table 2 performed with *lcc9*-antisense constructs, co-transformation rates were determined by integrated DNA from 66 randomly selected OK130 clones, through PCR amplification from genomic DNAs of *lcc9*-antisense fragments linked with *A. bisporus gpdII* promoter and *lcc1* terminator sequences using primers PF and PR (Table 5). Accordingly, 80 and 72% of the obtained clones were co-transformants of both plasmids. Functionality of inserted DNA in *lcc9*-silencing was then tested in co-cultivation of transformants in SAHX medium according to Pan et al. [65] with the fungus *Gongronella* sp. w5 which induces *lcc9* expression in OK130 [65, 67]. Using cDNAs from co-cultivated OK130 transformants and qRT-lcc9-F and qRT-lcc9-R as primers (Table 5), qRT-PCR analysis revealed silencing ratios of *lcc9* in 47% and 67% of all transformants for the two *lcc9* antisense constructs, respectively.

**Table 5:** Primers used in this study

Name	Sequence (5′-3′)	Purpose
ade8_f	*GAATTGGGTACCGGGCCCCCCCTCGAGGTCGAC*TGGCCGTTCATAGCGATGTC (sequence upstream of the *Hin*dIII-site in pRS426 in italic, sequence upstream of *ade8*^+^ in normal letters)	Cloning of *ade8*^+^ in p*Cc*Ade8
ade8_r	*GCCGCTCTAGAACTAGTGGATCCCCCGGGCTG*AGCTCGTTTCCATCGTCATCA (sequence downstream of the *Eco*RI-site in pRS426 in italic, sequence downstream of *ade8*^+^ in normal letters)	Cloning of *ade8*^+^ in p*Cc*Ade8
Lcc5-fwd	*CTCCCATCTACACACAACAAGCTTATCGCC*ATGTCGTTTGCTTGGAAAGCATTGGC (*A. bisporus* P_*gpd*_ sequence is in italic, *lcc5* sequence in normal letters)	Cloning of *lcc5* for overexpression in pYSK-*lcc5*
Lcc5-rev	*CCACTGGCCCTCTGGTCAACTATAATATTAT*TTAGGGATACATAGGGAGCAAGTTCGAA (T_*lcc1*_ sequence is in italic, *lcc5* sequence in normal letters)	Cloning of *lcc5* for overexpression in pYSK-*lcc5*
Lcc9-fwd	*CTCCCATCTACACACAACAAGCTTATCGCC*ATGTCCAGGAAACTTTTCTCTCTCGCC (*A. bisporus* P_*gpd*_ sequence is in italic, *lcc9* sequence in normal letters)	Cloning of *lcc9* for overexpression in pYSK-*lcc9*
Lcc9-rev	*CCACTGGCCCTCTGGTCAACTATAATATTAT*TTAAGGAGTGGGGACAATTTGGATAGAGGT (T_*lcc1*_ sequence is in italic, *lcc9* sequence in normal letters)	Cloning of *lcc9* for overexpression in pYSK-*lcc9*
Lcc9-antisense 1-fwd	*CTCCCATCTACACACAACAAGCTTATCGCC*CGGGATTCTCATAGTTGTAAGTGCTGC (*A. bisporus* P_*gpd*_ sequence is in italic, *lcc9* antisense 1 sequence in normal letters)	Cloning of *lcc9*-antisense fragment 1 in pYSK-*lcc9-*antisense-1
Lcc9- antisense 1-rev	*CACTGGCCCTCTGGTCAACTATAATATTAT*AGATGGGCCTTGGACCTGCCG (T_*lcc1*_ sequence is in italic, *lcc9* antisense 1 sequence in normal letters)	Cloning of *lcc9*-antisense fragment 1 in pYSK-*lcc9-*antisense-1
Lcc9- antisense 2-fwd	*CTCCCATCTACACACAACAAGCTTATCGCC*CGGACCACTTCCTCCTGGGGCA (*A. bisporus* P_*gpd*_ sequence is in italic, *lcc9* antisense 2 sequence in normal letters)	Cloning of *lcc9*-antisense fragment 2 in pYSK-*lcc9-*antisense-2
Lcc9- antisense 2-rev	*CACTGGCCCTCTGGTCAACTATAATATTAT*CTCTCATGGTCGACGAAATCCAGATC (T_*lcc1*_ sequence is in italic, *lcc9* antisense 2 sequence in normal letters)	Cloning of *lcc9*-antisense fragment 2 in pYSK-*lcc9-*antisense-2
P_*gpd*_-F	GATATCGAAGAAGAATTCAGAGGTCCGCAAGTA (*A. bisporus* P_*gpd*_ sequence, *Eco*RV site underlined)	Cloning of *A. bisporus gpdII* promotor for pCRII-*hph-lcc9* vector construction
P_*gpd*_-R	AAGTGGTCCG*GGCGATAAGCTTGTTGTGTGTAGATGG* (*A. bisporus* P_*gpd*_ sequence is in italic, *lcc9* antisense 2 sequence in normal letters)	Cloning of *A. bisporus gpdII* promotor for pCRII-*hph-lcc9* vector construction
Lcc9-antisense-hphF	*GCTTATCGCC*CGGACCACTTCCTCCTGGGGCA (*A. bisporus* P_*gpd*_ sequence is in italic, *lcc9* antisense 2 sequence in normal letters)	Cloning of *lcc9* antisense fragment 2 for pCRII-*hph-lcc9* vector construction
Lcc9-antisense-hphR	*TGCTATGACT*CTCTCATGGTCGACGAAATCCAGATC (*T*_*lcc9*_ sequence is in italic, *lcc9* antisense 2 sequence in normal letters)	Cloning of *lcc9* antisense fragment 2 for pCRII-*hph-lcc9* vector construction
T_*lcc9*_-F	ACCATGAGAG*AGTCATAGCACATAGCCATACCGACAC* (*T*_*lcc9*_ sequence is in italic, *lcc9* antisense 2 sequence in normal letters)	Cloning of *C. cinerea lcc9* terminator for pCRII-*hph-lcc9* vector construction
T_*lcc9*_-R	GGGCCCGTCAAAGGAGTCAGCCCTTGGACATG (*T*_*lcc9*_ sequence, *Apa*I site underlined)	Cloning of *C. cinerea lcc9* terminator for pCRII-*hph-lcc9* vector construction
DPf	ATGTCGATCCGCATCCTACTCCTC (sequence of *ade8* from startcodon onwards)	Diagnosis PCR for nuclear *ade8*^+^ insertion
DPr	ATCCCAGGCGGAGAGATTGCG (sequence of *ade8* with its last triplets for amino acids)	Diagnosis PCR for nuclear *ade8*^+^ insertion
PF	ACATCCACCATCTCCGTTTTCTCCCAT (*A. bisporus* P_*gpd*_ sequence)	PCR of OK130 co-transformants of *lcc9*-antisense-constructs
PR	TGACTATAGCAGCCTCCTACCACTG (T_*lcc1*_ sequence)	PCR of OK130 co-transformants of *lcc9*-antisense-constructs
qRT-lcc9-F	ATGTCCAGGAAACTTTTCTCTCTCG (*lcc9* sequence + 1 to + 25)	qRT-PCR of *lcc9*
qRT-lcc9-R	ATGTTCGAGACCGTCATGGTACT (reverse complementary *lcc9* sequence of + 79 to + 101)	qRT-PCR of *lcc9*

### The bacterial *hph* gene in OK130 transformations

We also used vector pCRII-hph with an integrated antisense-*lcc9* fragment for transformation of monokaryon OK130 under hygromycin B resistance selection. Transformation rates in 5 rounds of experiments were not as efficient, with only between 7 to 15 transformants per 1 µg plasmid DNA. After re-screening on new plates containing 200 mg/l hygromycin B, 40 of a total of 70 transferred clones (= 57%) failed to grow. Noteworthy, the tolerance of OK130 to hygromycin B varied among different batches of experiments. Screening under a constant hygromycin B concentration of 200 mg/l in the overlay on regeneration agar plates did not always work, leading sometimes to high proportions of false-positive transformants. Of the 30 remaining hygromycin B-resistant clones tested positive by PCR for *hph* integration, 12 (= 40%) were silenced for laccase Lcc9 production as determined by qRT-PCR analysis of cDNAs from transformants co-cultured with *Gongronella* sp. w5. In summary, *hph* selection and transformation efficiencies were inferior to the *ade8*^+^ selection and transformation efficiencies in OK130 with vector p*Cc*Ade8 while *lcc9* silencing frequencies in co-transformants were nearly as good.

## Conclusions

In this work, we have constructed p*Cc*Ade8 as a new selection vector for transformations of *C. cinerea* strains with *ade8* auxotrophies, such as the sequenced reference monokaryon OK130. Co-transformation rates of genes expressed from unselected vectors transformed with p*Cc*Ade8 were between 26 and 67% in ranges as observed in co-transformations with other selection markers in other strains. Using gene *ade8*^+^ for selection, this had no recognizable negative feedback effects on reducing numbers of viable transformants, similar as when using the *pab1*^+^ selection marker of *C. cinerea* for *pab1* complementations and unlike as experienced with the *trp1*^+^ selection marker in *trp1*-auxotrophic *C. cinerea* strains. *pab1*^+^ can be used to complement the *pab1-1* defect in the also sequenced homokaryon AmutBmut. Defects in the mutated *ade8-1* and *pab1-1* alleles in the two sequenced *C. cinerea* reference strains were defined as missense mutations in the N-terminal GARS domain of the bifunctional GARS-AIRS enzyme from the de novo purine biosynthesis pathway and in the C-terminal PabB domain of the bifunctional 4-amino-4-deoxychorismate synthase in the PABA biosynthesis pathway, respectively.

We have used *lcc9*-antisense constructs in co-transformation of strain OK130 with p*Cc*Ade8 in order to suppress native laccase production at high frequency in resulting transformants. Other attempts of *lcc9* silencing were made with a single vector carrying an *hph* selection marker and in addition cloned *lcc9*-antisense sequences for gene silencing. This second selection system is independent of a gene defect in a host strain. It is in principle also working, but was less efficient in transformation rates than using the p*Cc*Ade8 vector in single-vector transformation and in co-transformation. By its better transformation efficiency, *ade8*^+^ selection would thus be the first choice for transformation of the *C. cinerea* reference monokaryon OK130. Nevertheless, when further rounds of transformations in the same strain background are required, *hph* selection offers extra possibilities after a complementation of the *ade8-1* defect in OK130 by transfer of *ade8*^+^.

## Methods

### Strains, transformation and growth conditions

Monokaryons Okayama 7/#130 (short name in literature OK130 [8]; ATCC MYA-4618, FGSC 9003; genotype: *A43*, *B43*, *ade8-1*), FA2222 (DSM 28333; *A5*, *B6*, *acu1*, *trp1.1,1.6* [69]) and PG78 (DSM 28337; *A6*, *B42*, *pab1-1*, *trp1.1,1.6* [69]), and the self-fertile homokaryon AmutBmut (FGSC 25122; genotype: *A43mut*, *B43mut*, *pab1-1* [69]) were routinely cultivated on YMG/T medium at 37 °C [3]. Oidia per fully grown plates were harvested in sterile water, filtered through sterile glass wool, washed, protoplasted and transformed as described before [3, 4]. For fungal transformation, plasmid DNA with bacterial RNA was isolated from 3 ml *E. coli* XL1-Blue (Agilent, Böblingen, Germany) overnight LB (amp) cultures by a modified Birnboim-Doly method [4]. Per transformation sample and per plasmid, 1 µg plasmid DNA was used. When required for testing laccase activities in transformants, 0.5 mM ABTS was added to regeneration agar [16]. Prototrophic transformants appeared at first on regeneration agar 3.5–4 days after plating (= 1st day of picking clones reported in Tables 2,3,4). Day by day, all new clones were counted and collected from regeneration agar onto minimal medium with suitable supplements [3, 4]. Using in experiments the same protoplast batches, ratios of transformants were calculated by dividing the total number of clones obtained by a co-transformation by the total number of clones obtained from the single-vector transformation under the same scheme of selection. For selection for hygromycin B resistance after transformation, an extra 5 ml of regeneration agar (low melting point agar, 1%) containing 200 mg/l hygromycin B were overlaid after protoplast plating on regeneration agar. Individual hygromycin B-resistant transformants which appeared on these plates were re-screened by culturing again on regeneration agar containing 200 mg/l hygromycin B. *hph*-transformants were further verified based on PCR amplification of a *gpdII* promoter-*lcc9* antisense-*lcc9* terminator fragment with their genomes as templates and P_*gpd*_-F and T_*lcc9*_-R as primers (Table 5). OK130 transformants for *lcc9* silencing were cultured in SAHX medium using sucrose as the carbon source and cocultivation with *Gongronella* sp. w5 for *lcc9* induction according to Pan et al. [65]. qRT-PCR analysis using qRT-lcc9-F and qRT-lcc9-R as primers and transformants’ cDNAs as substrate was performed to further evaluate their silencing ratios [72]. For laccase activity tests in fermentation, clones were grown in YMG medium and supernatants of the culture broth were withdrawn every 12 h for activity assay and native-PAGE was performed as previously described [65]. Lcc1, Lcc5 and Lcc9 can be well distinguished in native-PAGE by differential migration patterns [64, 65].

### pCcAde8 vector construction

Chimeric primers ade8_f and ade8_r (Table 5) were designed from the AmutBmut genome for PCR amplification of the wt *ade8*^+^ gene from chromosomal DNA using Phusion High-Fidelity DNA polymerase (Thermo Fisher Scientific Inc., Darmstadt, Germany). The amplified DNA fragment was transformed into the *∆ura3* yeast strain RH 1385 [70] together with the *Hin*dIII-*Eco*RI double-digested *E. coli*-yeast shuttle *ura3*^+^-vector pRS426 [60] for in vivo plasmid construction by homologous recombination [71]. Plasmids were isolated from prototrophic yeast clones and further amplified in *E. coli* XL1-Blue. Proper fragment insertion was confirmed by sequencing as described [6]. Diagnosis PCR for insertion of p*Cc*Ade8 in nuclear DNA of transformants was performed with primers DPf and DPr (Table 5) which amplify the complete *ade8* coding region. Sequencing of the amplicons from 25 randomly selected transformants verified insertion of *ade8*^+^ copies by presence of either a wt A (1x) or a mixture of an A and a mutant G (24x) at position 691 in codon 231 of the gene.

### Other plasmids

*trp1*^+^-vector pBD5 and *trp3*^+^*-*vector pDB3 are described in [5] and [7], respectively. pPAP1-2 is a pTZ18R-based *pab1*^+^ selection vector [3]. Plasmid pYSK7 is a pRS426 [60] derivate containing the *C. cinerea* laccase gene *lcc1* cloned behind the *A. bisporus gpdII* promoter and with its own terminator [16]. pYSK-*lcc5* and pYSK-*lcc9* were generated through in vivo recombination in yeast [71] of PCR-amplified OK130 cDNA (for primers, please see Table 5) with *Bam*HI and *Hpa*I linearized plasmid pYSK7. Similarly, pYSK-*lcc9-*antisense-1 and pYSK-*lcc9*-antisense-2 were constructed by amplifying *lcc9* sequences with primers Lcc9-antisense 1/2-fwd and Lcc9-antisense 1/2-rev (see Table 5) from strain OK130 and inserting the resulting fragments (*lcc9*-antisense 1 is from bp + 305 to + 514 of *lcc9*; *lcc9*-antisense 2 is from bp + 752 to + 1032 of the gene) into *Bam*HI and *Hpa*I linearized plasmid pYSK7 through in vivo recombination in yeast [71]. The *lcc9*-antisense 2 plasmid pCRII-*hph-lcc9* was constructed based on the pCRII-TOPO derivative pCRII-*hph* which contains in the vector TOPO TA-cloning site a 1.0 kb β-tubulin promoter and a 0.5 kb terminator sequence of *Trametes hirsuta* AH28-2 and the bacterial *hph* gene in between [72]. Briefly, a 281 bp reverse complementary sequence cloned from cDNA of laccase gene *lcc9* (bp + 752 to + 1032) was joined to the *A. bisporus gdpII* promoter sequence (277 bp) and the *C. cinerea lcc9* terminator sequence (500 bp) by overlapping PCR using the primer pairs of P_*gpd*_-F and P_*gpd*_-R, and T_*lcc9*_-F and T_*lcc9*_-R listed in Table 5. The fused sequences were then digested with *Eco*RV and *Apa*I and inserted into the *Eco*RV and *Apa*I polylinker sites of pCRII-*hph*.

### Sequence analyses

The published genomes of monokaryon Okayama 7/#130 (https://mycocosm.jgi.doe.gov/Copci1/Copci1.home.html) and homokaryon AmutBmut (https://mycocosm.jgi.doe.gov/Copci_AmutBmut1/Copci_AmutBmut1.home.html) on the JGI Mycocosm side were used for defining chromosomal loci of genes of interest and obtaining relevant DNA and protein sequences. Protein sequences from *E. coli* and *S. cerevisiae* (Table 1) were used in tblastn searches. Homologous protein sequences retrieved from the JGI homepages and from NCBI were aligned by ClustalX 2.0 [73] and the MEGA 6.0 software was used with 1000 bootstrap values for constructing a neighbor-joining tree [74].

## Data Availability

No larger data sets were generated and analyzed during this study. Vectors are available from the authors.

## Abbreviations

ABTS: 2,2′-Azino-bis (3-ethylbenzothazoline-6-sulfonic acid)
ADC: 4-Amino-4-deoxychorismate
ADP: Adenosine diphosphate
AICAR: 5´-Phosphoribosyl-5-monophosphate
AIR: Aminoimidazole ribonucleotide
AIRS: Aminoimidazole ribonucleotide synthase
AMP: Adenosine monophosphate
ATP: Adenosine triphosphate
CAIR: 5-Amino-4-imidazolecarboxamide ribonucleotide
DHF: Dihydrofolic acid
DHNTP: 7,8-Dihydroneopterin 3′-triphosphate
DHP: Dihydropteroate
FAICAR: 5-Formamidoimidazole-4-carboxamide ribotide
FGAM: Formylglycinamide ribonucleotide
FGAMS: Formylglycinamide ribonucleotide synthase
FGAR: Phosphoribosyl-*N*-formylglycinamide
GAR: Glycinamide ribonucleotide
GARS: Glycinamide ribonucleotide synthase
GART: Phosphoribosylglycinamide formyltransferase
GATase: Glutamine amidotransferase
GPAT: Glutamine amidophosphoribosyltransferase
GTP: Guanosine-5′-triphosphate
HIT: Histidine triad
IGP: Indole-3-glycerol-phosphate
IGPS: Indol-3-glycerol-phosphate synthase
IMP: Inosine monophosphate
NAD: Nicotinamide adenine dinucleotide
NADP: Nicotinamide adenine dinucleotide phosphate
3PHP: 3-Phosphohydroxypyruvate
PABA: *para*-Aminobenzoic acid
PAGE: Polyacrylamide gel electrophoresis
3PG: 3-Phosphoglyceric acid
Pi: Phosphate
PRA: Phosphoribosylamine
PRAI: Phosphoribosylanthranilate isomerase
PRPP: 5-Phosphoribosyl-α-1-pyrophosphate
SAICAR: Phosphoribosylaminoimidazole-succinocarboxamide
SAICARS: Phosphoribosylaminoimidazole-succinocarboxamide synthase
SDH: Succinate dehydrogenase
SAMP: Succinyladenosine 5′-monophosphate
THF: Tetrahydrofolate
wt: Wildtype
